# Weight loss with or without intragastric balloon causes divergent effects on ghrelin cell expression

**DOI:** 10.1002/osp4.478

**Published:** 2021-01-13

**Authors:** Elisabeth Mathus‐Vliegen, Anna Spångeus, Susanna Walter, Ann‐Charlott Ericson

**Affiliations:** ^1^ Department of Gastroenterology and Hepatology Academic Medical Centre (AMC) University of Amsterdam Amsterdam the Netherlands; ^2^ Department of Health, Medicine and Caring Sciences Division of Diagnostics and Specialist Medicine Linköping University Linköping Sweden; ^3^ Department of Acute Internal Medicine and Geriatrics Linköping University Hospital Linköping University Linköping Sweden; ^4^ Department of Biomedical and Clinical Sciences Division of Inflammation and Infection Medical Faculty Linköping University Linköping Sweden; ^5^ Department of Gastroenterology Linköping University Hospital Linköping University Linköping Sweden; ^6^ Department of Biomedical and Clinical Sciences Division of Molecular Medicine and Virology Medical Faculty Linköping University Linköping Sweden

**Keywords:** ghrelin cell, grelin, ghrelin secretion, immunohistochemistry, intragastric balloon

## Abstract

**Objective:**

The mechanism of action of intragastric balloons in the treatment of obesity is not fully understood. One of the hypotheses is that balloons might have an effect on the fundus, the area of ghrelin production.

**Methods:**

Participants were randomized to a 13‐week period of sham or balloon treatment followed by a 13‐week period of balloon therapy in everyone. Blood samples for ghrelin levels were taken in the fasting state and after a breakfast at the start, after 13 and 26 weeks. Biopsies for ghrelin cell immunohistochemistry were taken from the fundus at endoscopy.

**Results:**

Seven participants entered the balloon–balloon (BB) group and 11 the sham–balloon (SB) group. Despite a considerable weight loss, a median −17.9 kg (interquartile ranges −23.8 to −0.5) in the BB group and −18.3 kg (−22.7 to −14.7) in the SB group, fasting ghrelin and meal‐induced ghrelin response did not change. In the SB group, the number of ghrelin cells increased significantly (*p* 0.001) from 110.6 (83.6–118.9) to 160.2 (128.5–223.0) while on sham treatment and returned to initial levels, 116.3 (91.7–146.9) (*p* 0.001), when they received their first balloon. No significant changes in ghrelin cell numbers were observed in the BB group.

**Conclusion:**

In participants without a balloon, weight loss induced an increase in ghrelin cell numbers in the fundus, which was annulled by the subsequent placement of a balloon. The effect of a balloon might be explained by effects on ghrelin cell numbers or ghrelin cell activity.

## 1 INTRODUCTION

The treatment of obesity consists of a stepwise approach of combined lifestyle intervention, a combination of energy restriction, physical exercise and behavioral modification, followed by drug therapy and surgery.^1^ ^,^ ^2^ To date, bariatric surgery is the most effective intervention strategy resulting in sustained body weight loss and reduction of comorbidities.^3^ For those who do not respond to medical therapy or are not or not yet surgical candidates or refuse surgery because of its invasiveness and fear of complications, less invasive endoscopic treatments might look attractive. These treatments, also called Endoscopic Bariatric and Metabolic Therapy, include intragastric balloon systems.^4^, ^5^, ^6^, ^7^, ^8^ Promising long‐term weight maintenance data have been reported with this method.^9^ ^,^ ^10^ In patients with super‐obesity, preoperative treatment with intragastric balloons considerably reduced liver volume to facilitate surgery^11^ and decreased operative time and overall risk of significant adverse outcomes.^12^ Meanwhile, two balloons have been approved by the FDA for a 6‐month therapy in patients with a BMI 30–40 kg/m^2^ with the requirement of supportive therapy for a total of 12 months: that is; (1) the Orbera balloon (formerly the BioEnterics Intragastric Balloon or BIB, Apollo Endosurgery) since 2015^13^ ^,^ ^14^; and (2) the Obalon orally ingested intragastric balloon (Obalon Therapeutics) since 2016.^15^ ^,^ ^16^ Pivotal trials with two new and innovative balloon systems have just been finished and await approval by the FDA.^17^, ^18^, ^19^

However, the exact mechanism by which intragastric balloons induce weight loss is still not known. Intragastric balloons are hypothesized to mediate satiety *peripherally*, by being a physical impediment of food intake, by reducing the gastric capacity and by delaying gastric emptying, and *centrally*, by activating gastric stretch receptors that transmit signals via afferent vagal nerves, the solitary tract and paraventricular nuclei, to the ventromedial and lateral hypothalamus.^4^ ^,^ 20, 21, 22 Short‐term satiety is primarily affected by gastric distension and gastric volume, that is, by the weight and volume of the food rather than its energy content.^22^ ^,^ ^23^ This volume‐regulated satiety is thought to result primarily from gastric distension. Indeed, mechanical gastric balloon distension to a volume greater than 400 ml during meals significantly reduced oral intake.^23^ ^,^ ^24^

After the intake of a meal the gastrointestinal tract releases regulatory peptide hormones contributing to the feeling of satiety.^20^ The release of postprandial hormones is principally mediated by two mechanisms; (1) by the contact of nutrients, with its different physiochemical properties with receptors in the GI tract; and (2) by the mechanical distension of the stomach leading to increased neural afferent firing in the vagal nerve, affecting feeding‐related areas in the brain, including the hypothalamus.^4^ ^,^ ^21^

To date there are only few prospective studies evaluating the effect of chronic gastric distension on neuroendocrine mechanisms in humans. Mathus‐Vliegen et al. reported the effects of sham treatment or balloon positioning on gut hormones such as cholecystokinin (CCK) and pancreatic polypeptide (PP).^25^ Ghrelin is an orexogenic hormone in contrast to CCK and PP. It is secreted by the fundic glands of the stomach and usually increases food intake.^26^ Receptors for ghrelin are expressed on capsaicin sensitive vagal afferent neurons innervating the gut.^27^ In animal experiments it has also been shown that gastric distension affects the leptin sensitivity of the nucleus tractus solitarius.^28^

Fasting ghrelin levels have been investigated in patients treated with air‐filled and fluid‐filled balloons. An 800‐ml air‐filled balloon resulted in increased ghrelin levels after 1 and 4 weeks of balloon insertion and decreased at the time of balloon removal after 16 weeks.^29^ Most studies have been performed with a 500–700 ml fluid‐filled balloon with divergent results. Fasting ghrelin levels have been reported to remain unchanged^30^ or to increase^29^, ^30^, ^31^ during intragastric balloon therapy. Mion et al. reported that chronic gastric dilatation with a balloon reduced fasting plasma ghrelin levels, indirectly indicating that chronic gastric distension might have an inhibitory effect on ghrelin secretion.^32^ They also demonstrated a significant decrease in gastric emptying rates.

There are only a few studies that compared balloon treatment with a control group. Konopko‐Zubrzycka et al. found a significant increase in fasting ghrelin levels after 1 month and 6 months of balloon therapy compared with controls, but in the former group weight losses were 4 to 5 times as great.^33^ Martinez‐Brocca et al. found similar weight losses in balloon‐treated and control patients and no differences between the groups in fasting and postprandial ghrelin levels.^34^ Mathus‐Vliegen et al. performed a patient‐ and physician‐blinded sham‐controlled balloon treatment study and found that both balloon‐balloon and sham‐balloon treatment resulted in significant weight losses after 13 and 26 weeks, but unexpectedly, fasting ghrelin concentrations and the ghrelin response after a meal did not change and certainly did not rise, as is usually seen after energy restriction and weight loss.^35^ ^,^ ^36^ When the balloon was located in the fundus region, ghrelin concentrations were more suppressed.^35^

The mode of action of intragastric balloons could thus partly be ascribed to the inhibition of ghrelin secretion, that is, more specifically when the balloon was located in the fundus where ghrelin is secreted. Therefore, we decided to use biopsies taken from the fundus to explore whether weight loss with an intragastric balloon compared with sham treatment was associated with a change in the number of ghrelin cells.

## 2 PATIENTS AND METHODS

### 2.1 Study design

The study design has been described in detail.^5^ ^,^ ^25^ ^,^ ^35^ In brief, participants involved in a trial in the Academic Medical Centre of the University of Amsterdam, investigating the 1‐year weight loss with intragastric balloon treatment (Orbera, Apollo Endosurgery) and the weight maintenance in a 1‐year balloon‐free follow‐up period were asked to take part in a study investigating the mechanism of action of intragastric balloons. Before entering the trial, participants had to conform to the criteria of eligibility, that is, age ≥18 years, BMI of at least 32 kg/m^2^ without significant changes over the last 3 months, and failure to lose weight within a supervised weight loss program. Also, gastrointestinal lesions and a large hiatal hernia (assessed at the screening endoscopy) and previous intra‐abdominal or bariatric surgery had to be absent.

**FIGURE 1:**
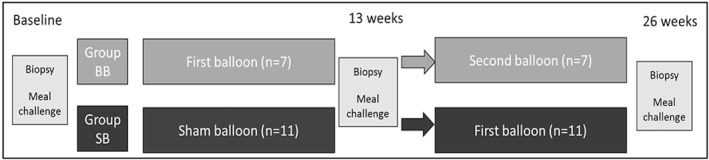
The study was designed as a 13 weeks double‐blind sham‐controlled period followed by a 13 weeks of balloon period for all participants. A total of 7 participants received balloon–balloon treatment (BB) and 11 participants had a sham period followed by a balloon period (SB). At time point 0, week 13 and week 26, participants underwent an endoscopy with biopsies and performed a meal challenge followed by blood sample testing

As shown in Figure 1, the trial was designed as a 13‐week double‐blind sham‐controlled period wherein neither the participant nor the endoscopist could perceive a difference between sham control and active balloon placements. Each participant underwent a screening endoscopy and the distance between incisor teeth and gastro‐esophageal junction was measured. Three biopsies were taken from the fundus and antrum and investigated for pathology and the presence of *Helicobacter pylori*, and eventual spread of *H. pylori* upwards to the fundus during balloon treatment. Biopsies from the roof of the fundus were taken in retroflexion and saved for immunohistochemical studies. After removal of the endoscope, the balloon placement assembly consisting of a sheath with a collapsed balloon and a balloon fill tube was inserted 10 cm distal to the gastro‐esophageal junction. The balloon was filled with 500 ml saline via the fill tube and upon removal of the fill tube the balloon valve closed. In case of the sham treatment, the collapsed balloon was not present in the sheath but the same resistance was felt upon inflation, and the stomach was filled with 500 ml saline.

In the second period of 13 weeks, all participants received a balloon. After each balloon placement and also each time the balloon was exchanged or removed, a plain X‐ray in standing position was made to confirm the proper positioning of the balloon after placement and its location in the stomach before removal. This information was kept blinded to the endoscopists. Biopsies from the gastric antrum and fundus were taken during the endoscopy procedure at week 0 (T0), 13 (T13), and 26 (T26).

At the start, after 13 weeks and after 26 weeks, the participants fasted from midnight and came to the hospital at 8.30 AM. After two fasting samples of blood they received a breakfast meal consisting of a pancake with butter and jelly and 150 ml orange juice (2.8 MJ [672 kcal] with 79 g carbohydrates [47% kcal; 47% of total energy], 31 g fat [42% kcal], and 19 g protein [11% kcal]). The pancakes were prepared elsewhere to avoid influences of smell, sight, and food expectation. The fasting samples and samples taken after 15, 30, 45, and 60 min after the breakfast meal were centrifuged and plasma was stored at −70°C. Because samples were collected and stored without acidification to stabilize the labile side chain of the acylated ghrelin, total plasma ghrelin was analyzed. Under a wide variety of physical condition the ratio of acylated and total ghrelin is constant and thus total ghrelin is a reasonable surrogate for acylated ghrelin.^37^ Total plasma ghrelin was measured using a commercially available RIA kit (Linco Research Inc.). It has a 100% specificity for ghrelin and a limit of sensitivity at 93 pg/ml. The mean intra‐assay coefficient of variation was 5.7% (range, 2.4–8.4) at the lower concentration (353–734 pg/ml) and 3.7% (range, 1.0–9.0) at the higher concentration (905–1.879 pg/ml).^35^ All samples were measured in duplicate and in one run.

### 2.2 Participants

**FIGURE 2:**
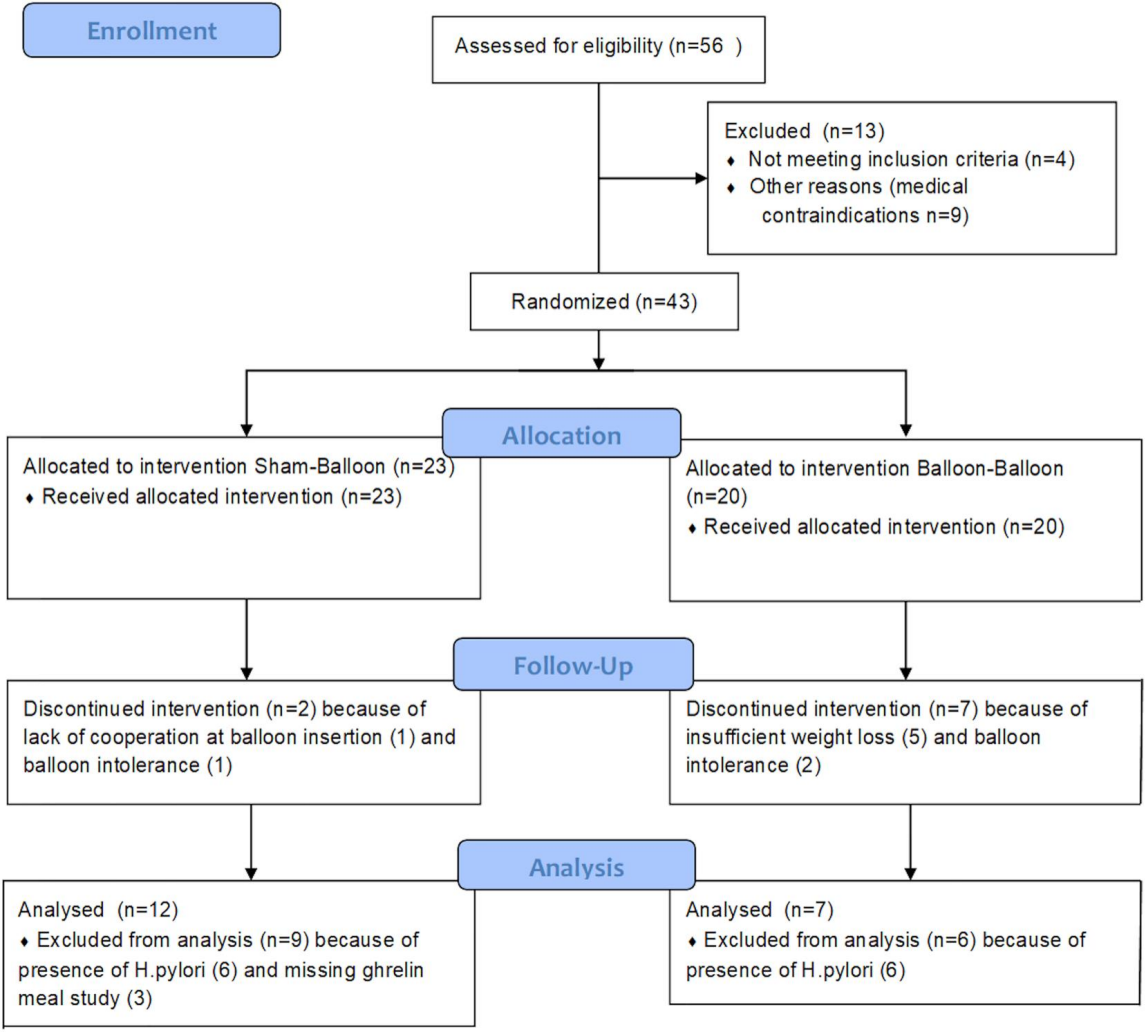
CONSORT flow diagram for the ghrelin study

The present study comprises all 43 participants from the original trial with complete biopsies and blood ghrelin values from the three time‐points. Another requirement was the absence of *Helicobacter pylori* because of reported interference with plasma ghrelin levels and ghrelin immunoreactive cells.^38^, ^39^, ^40^, ^41^ Participants were withdrawn because of intolerance of the balloon or not achieving the per‐protocol required weight loss of 6.5 kg after the first 13 weeks of balloon treatment. This minimum weight loss was required by the FDA for safety and effectiveness reasons in this FDA approved protocol (Figure 2). A total of nineteen participants were included, seven participants belonged to the balloon–balloon (BB) group and twelve participants to the sham–balloon (SB) group.

### 2.3 Immunohistochemical procedures

Paraffin embedded biopsies from the 19 participants that participated during the whole study time of 26 weeks were prepared for the immunohistochemical analysis at the Division of Molecular Medicine and Virology, Linköping University, Sweden. Biopsies from T0, T13, and T26 were cut at 5 µm on a microtome. One subject from the SB group was later excluded due to the absence of glandular tissue in the biopsies.

The deparaffinization/rehydration procedure was performed by first heating the slides at 65°C for one hour followed by treatment in Tissue Clear (5 min; Sakura Finetek Europe BV) and subsequent rinsed in a series of descending concentrations of ethanol (absolute alcohol, 95%, 70%, water and finally tris‐buffered saline with Tween 20 [TBST, pH 7.6]).

Heat induced epitope retrieval was performed by treatment in sodium citrate buffer, pH 6.0, for 20 min. The slides were allowed to cool down and rinsed in TBST.

To block endogenous peroxidase, tissue sections were pretreated with Peroxidazed 1 (Biocare Medical) followed by the treatment with Background Sniper (Biocare Medical) to reduce nonspecific background staining. Sections were incubated in primary antiserum, mouse anti ghrelin (Abcam anti‐ghrelin antibody ab57222 [Abcam]), 1:100 in tris‐buffered saline (TBS) pH 7.6, overnight at room temperature. Sections were then incubated in biotinylated goat anti mouse antisera (Vector laboratories Ltd) 1:150 in TBS for one hour at room temperature. To visualize the biotinylated antisera, Vectastain^®^ ABC kit and DAB substrate kit (Vector laboratories Ltd) were used according to the experimental protocols provided from manufacturer. The incubations in antisera and ABC kit were followed by rinses in TBST (3 × 3 min) and treatments with DAB substrate kit were followed by a wash in tap water before being counterstained with Mayer's hematoxylin, dehydrated and cover slipped. The light microscopic analysis was performed in a Nikon Eclipse E800 light microscope (Nikon Instruments Europe BV). Negative controls were performed to rule out the possibility of nonspecific binding of secondary antisera to the tissue. In order to do that, sections without primary antisera were treated according to the immunohistochemical protocol and analyzed.

Unbiased microscopical analyses were performed by coding the slides. From each biopsy, three sections (50 µm in between) were analyzed. For each section three fields of view from the glandular part of the mucosa were examined by counting DAB‐labeled cells with visible nuclei. To obtain a correct area of the fields of view, the objective of the microscope was calibrated according to the imaging software used (Nikon Instruments Europe BV).

### 2.4 Statistical analysis

The effects of treatment were tested by Student *t* test between the groups and by paired *t* test within the same group for normally distributed variables. For non‐normally distributed variables, the Mann Whitney U test and the Wilcoxon signed‐rank test were used, respectively. The release of ghrelin during the meal was calculated as the area under the curve (AUC) using the trapezoidal rule (measured 15 min before meal until 60 min after meal). A *p* value < 0.05 was considered significant. Participant characteristics are given as median (and interquartile [IQR] ranges).

### 2.5 Ethics

The study was approved by the Medical Ethical Committee of the Academic Medical Centre, University of Amsterdam, the Netherlands for the first 13 weeks. Thereafter, by amendment, a second period of 13 weeks was allowed.

## 3 RESULTS

### 3.1 Participant characteristics

Participants did not differ as to age, body weight, BMI, fasting gastrin, and insulin levels (Table 1). In follow‐up, weight losses were substantial and did not differ between the two groups at 13 and 26 weeks. However, the second period of 13 weeks (week 13 to week 26) when the SB group received their first balloon while BB group continued with balloon therapy, revealed a significantly greater weight loss for the sham‐balloon group, that is, −9.0 (−10.8 to −5.2) versus −5.0 (−7.2 to −3.1) kg (*p* 0.027) (Table 1).

**TABLE 1:** Descriptive characteristics, and changes over time in the sham–balloon group and balloon–balloon group

		Sham–Balloon	Balloon–Balloon	*p*‐value
Baseline T0	Number (male/female)	11 (0/11)	7 (1/6)	
	Age	43 [39–47]	42 [3055]	*ns*
	Body weight kg	113.7 [97.9–119.3]	115.9 [101.5–123.8]	*ns*
	BMI (kg/m^2^)	39.2 [35.7–43.1]	42.6 [38.4–43.8]	*ns*
	Excess weight (%)	180 [15,564–196]	190 [169–200]	*ns*
	Gastrin mcg/L	0.02 [0.02–0.03]	0.02 [0.02–0.04]	*ns*
	Insulin mE/L	17 [12–30]	18 [11–56]	*ns*
	Preprandial/fasting ghrelin^a^(pg/mL)	670 [625–922]	654 [638–941814]	*ns*
	AUC ghrelin	49,820 [47,801–62,582]	43,998 [42,898–51,852]	*ns*
	Number of ghrelin cells/surface area	110.6 [83.6–118.9]	111.7 [84.8–189.0]	*ns*
13 weeks T1	Body weight kg	100.8 [91.0–109.9]	103.0 [88.3–107.4]	*ns*
	BMI (kg/m^2^)	35.4 [31.8–40.8]	37.5 [34.4–42.0]	*ns*
	Excess weight (%)	160 [145–180]	169 [151–184]	*ns*
	Weight loss kg first 13 weeks	−11.2 [−12.6 to −7.5]	−13.2 [−17.6 to −8.1]	*ns*
	Gastrin mcg/L	0.03 [0.02–0.04]	0.02 [0.02–0.04]	*ns*
	Insulin mE/L	14 [10–18]	15 [11–32]	*ns*
	Preprandial/fasting Ghrelin^a^(pg/mL)	765 [573–1,107,830]	691 [598,608–1019]	*ns*
	AUC ghrelin	53,231 [45,339–58,247]	52,521 [44,243–64,767]	*ns*
	Number of ghrelin cells/surface area	160.2 [128.5–223.0]	137.3 [63.1–173.3]	*ns*
26 weeks T2	Body weight kg	89.0 [83.5–102.0]	98.0 [87.4–100.2]	*ns*
	BMI (kg/m^2^)	32.6 [30.0–37.8]	36.0 [32.1–39.4]	*ns*
	Excess weight (%)	146 [136–166]	160 [142–171]	*ns*
	Weight loss kg week 13–26	−6.90 [−10.8 to −5.2]	−5.0 [−7.2 to +3.1]	*0.027*
	Weight loss kg overall after 26 weeks	−18.3 [−22.7 to −14.7]	−17.9 [−23.8 to −0.50]	*ns*
	Gastrin mcg/L	0.02 [0.02–0.03]	0.02 [0.02–0.06]	*ns*
	Insulin mE/L	13 [9–17]	15 [9–24]	*ns*
	Preprandial/fasting Ghrelin^a^(pg/mL)	822 [573,656–931,870]	763 [588–857]	*ns*
	AUC ghrelin	59,363 [45,442–65,868]	54,939 [42,632–63,670]	*ns*
	Number of ghrelin cells/surface area	116.3 [91.7–146.9]	102.6 [77.1–136.3]	*ns*

*Note*: Values are given as median and interquartile ranges.

^a^ 15 min before presenting the meal.

### 3.2 Immunohistochemistry of ghrelin cells

**FIGURE 3:**
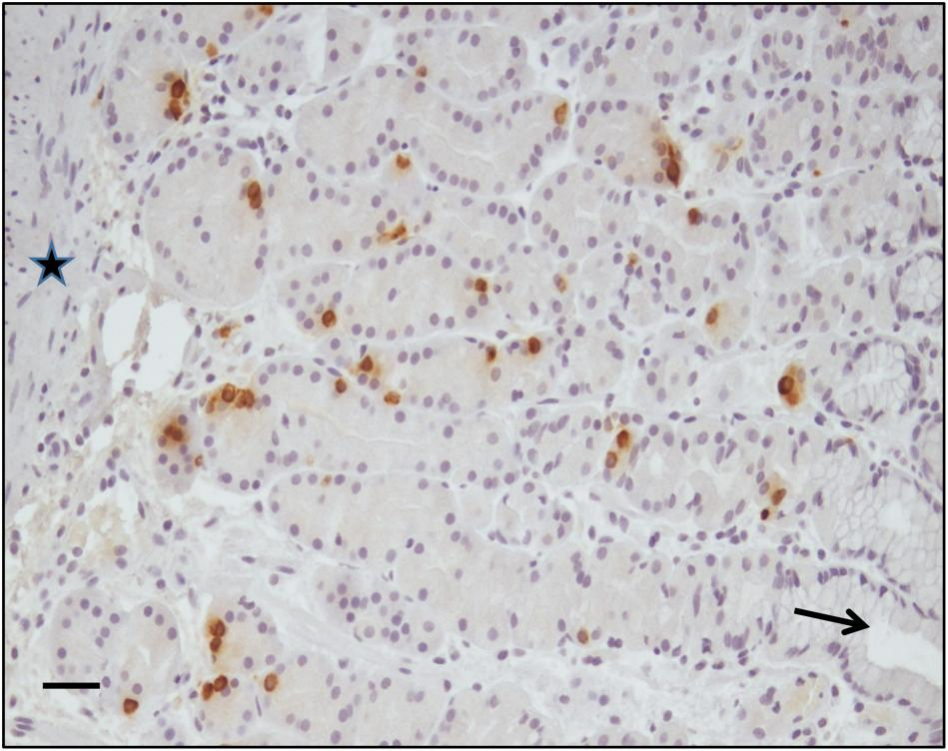
Brown DAB‐stained ghrelin immunoreactive cells are seen distributed in the glandular region of the fundus mucosa. Arrow point at a gastric pit in the apical region of the mucosa and the star indicates the muscularis mucosae at the basal part. Scale bar: 50 µm

**FIGURE 4:**
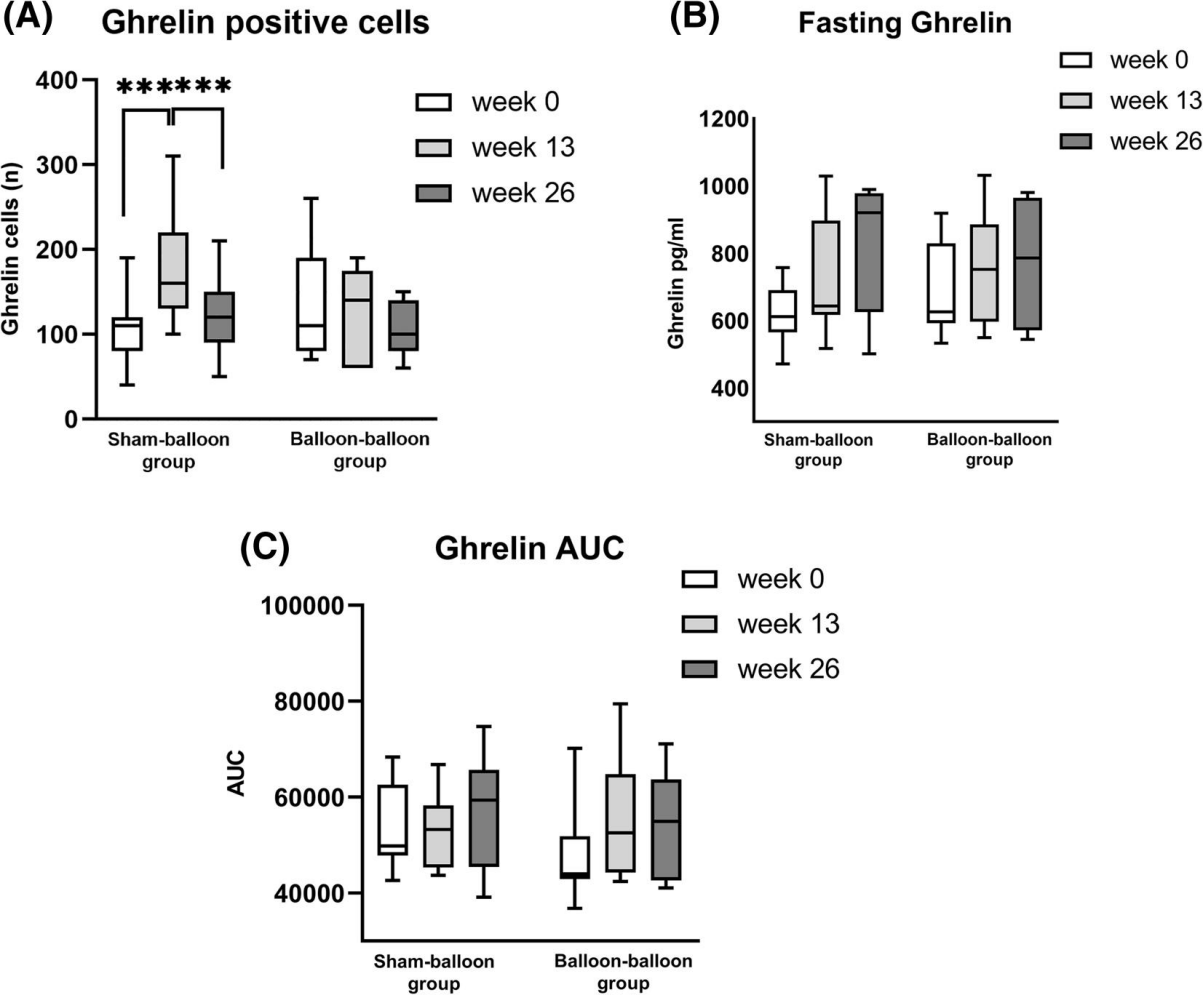
(A) Box‐and‐Whisker plot diagram of the number of ghrelin positive cells in the fundus at the start, after 13 and after 26 weeks in the sham–balloon group and the balloon–balloon group. There were no differences between the groups. Within the groups, the number of ghrelin cells increased significantly (*p* 0.001) during the period of sham treatment in the sham‐balloon group and decreased significantly (*p* 0.001) during their first balloon treatment. *** signifies *p* 0.001. (B) Box‐and‐Whisker plot diagram of fasting ghrelin levels (pg/ml) at the start, after 13 and after 26 weeks in the sham–balloon group and the balloon–balloon group. No differences were seen between and within groups. (C) Box‐and‐Whisker plot diagram of the meal‐related ghrelin secretion expressed as area under the curve of ghrelin secretion for 1 h at the start, after 13 and after 26 weeks in the sham–balloon group and the balloon–balloon group. No differences were seen between and within groups

Ghrelin immunoreactive cells were, as expected, seen distributed in the epithelium of the glandular portion in the fundus mucosa (Figure 3). The number of ghrelin positive cells did not differ between SB and BB group at inclusion or at time point 13 weeks or 26 weeks (Table 1). In contrast, when analyzing data within the groups, participants in the SB group showed a significantly higher number of ghrelin cells after 13 weeks (after sham period) as shown in Figure 4A (from 110.6 [83.6–118.9] to 160.2 [128.5–223.0] cells/surface area, *p* 0.001). However after having a balloon (i.e., after balloon at week 26) the number of cells decreased again (to 116.3 [91.7–146.9] cells/surface area, *p* 0.001). On the contrary participants in BB group did not show any significant alteration of number of ghrelin cells during the study period. Ghrelin cell numbers did not correlate with the position of the balloon on a plain X‐ray in standing position before removal.

### 3.3 Blood analysis of ghrelin

There was no difference between SB and BB regarding preprandial fasting ghrelin levels or meal‐suppressed ghrelin secretion (AUC ghrelin) at baseline or at the follow‐up period at week 13 and 26 (Table 1). Similarly no differences were seen over time, analyzing SB and BB group separately (Figures 4B,C). There was also no trend toward a significant increase (all *p* values above 0.2) in fasting and meal‐suppressed ghrelin secretion despite a visually suggestive gradually increasing course.

Nine participants had a BMI below 40 kg/m^2^ (38.4 [35.0–39.0]) and nine a BMI ≥ 40 kg/m^2^ (43.8 [42.7–46.8]). There were no differences in fasting ghrelin levels at the start, after 13 and 26 weeks between participants with less severe or severe obesity.

## 4 DISCUSSION

In a previous study Mathus‐Vliegen et al. demonstrated that the expected increase in ghrelin after a balloon‐induced weight loss of 15% did not occur,^35^ which has been noticed after weight losses as small as 5% of body weight.^36^ Specifically, when the balloon was located in the fundus region, the place of synthesis of ghrelin, ghrelin concentrations were more suppressed.^35^ Thus it was a logic extension of that finding to explore the number of ghrelin cells in this region. The hypothesis was that the continuous contact of ghrelin cells in the fundus with food on top of the balloon or food remaining in the stomach for a longer time due to delayed gastric emptying would decrease the need for ghrelin secretion or ghrelin cells. Indeed, in the presence of a similar weight loss, ghrelin cells did not change in participants treated with a balloon whereas ghrelin cells increased significantly in sham‐treated participants and decreased significantly to numbers at the start when these participants received their first balloon in the second period. This absence of ghrelin cell increase with a balloon might be of benefit as increased ghrelin levels after a period of negative energy balance and the hereby introduced weight loss may be responsible for an increased appetite and increased food intake which hinders further body weight loss and promotes weight regain. In contrast to the more extensive AUC ghrelin study where there was a greater influence on ghrelin secretion when the balloon was in the fundus, we could not find such a correlation as to ghrelin producing cells in the present study.^35^ Only 38% of balloons were truly located in the fundus, the remainder was either in the mid stomach or in the antrum.

To our knowledge, this finding of increased ghrelin producing cells in participants losing weight is a new finding. Maksud et al. compared patients with severe obesity with normal‐weight dyspeptic patients and found a higher density of ghrelin producing cells in patients with severe obesity.^42^ However, they did not address the fate of these cells after weight loss. Goitein et al. investigated sleeve gastrectomy specimens and discovered a decline in average ghrelin cell counts from fundus to the pre‐antral region.^43^ In a study, using the articulating circular endoscopic stapler to perform a gastroplication transorally, we found a median excess weight loss of 34.9% (IQR 17.8 – 46.6).^44^ A substudy with biopsies of the fundus, antrum and duodenum before and one year after the procedure revealed a downregulation of MBOAT4 (Membrane‐bound O‐Acyl‐Transferase domain containing 4), the gene encoding for the ghrelin activating enzyme GOAT (Ghrelin‐O‐Acyl‐Transferase) and a trend for downregulation of ghrelin expression itself.^45^ Unfortunately, a counting of fundus ghrelin cells was not performed.

Most of the studies that tried to explain the mechanism of action of intragastric balloons focused on gastric emptying. Interestingly, in this respect is the finding that the degree of weight loss was highest in participants who had the greatest delay in gastric emptying after balloon positioning.^46^, ^47^, ^48^ It would have been interesting to measure fasting and meal‐suppressed ghrelin levels in these participants to substantiate our findings.

The strengths and limitations of this study should be acknowledged. The strength of this study is that the study design allowed to study the consecutive effects of sham treatment and balloon positioning in the SB group and the effects of twice balloon therapy in the BB group. The small number of participants remaining after exclusion of those with *H. pylori* and of those who did not have 3 consecutive fasting and meal‐suppressed ghrelin values or representative biopsies is certainly a limitation. Unexplained is the fact that we did not observe an increase in fasting ghrelin levels or a reduced postprandial ghrelin inhibition as a function of a higher number of ghrelin cells in the sham group, which either might indicate a lesser secretory activity of the ghrelin cells or might be due to insufficient statistical power. This study could, however, not substantiate the finding of Nicolic et al. who suggested that participants with severe obesity are ghrelin hyporesponders as participants with less severe and severe obesity demonstrated similar fasting ghrelin levels.^30^ Also, we did not measure gastric emptying, but two findings indicated a delayed gastric emptying: the decreased meal‐stimulated CCK release^25^ and the finding of food obstructed above or adhered to the balloon as could be judged at endoscopy upon balloon exchange.^5^

## 5 CONCLUSION

In conclusion, this study showed that weight loss in those without a balloon, that is, the sham‐treated group, induced an increase in ghrelin cell numbers in the fundus, in contrast to those who lost weight with having a balloon where no such ghrelin cell increase was seen. To what extent the degree of prolongation of the gastric emptying time and its prognostic value as to weight loss are explained by effects on ghrelin release and ghrelin cell numbers or ghrelin cell activity should be worthwhile to investigate further.

## CONFLICT OF INTEREST

None of the authors has any conflict of interest.

## AUTHOR CONTRIBUTIONS

Eisabeth Mathus‐Vliegen conceived and carried out the experiment; Ann‐Charlott Ericson carried out the immunohistochemistry work; Eisabeth Mathus‐Vliegen, Anna Spångeus, Susanna Walter and Ann‐Charlott Ericson analyzed the data; and all authors were involved in writing the paper and approved the final paper as has been submitted for publication.

## CHECKLIST

The Consort Checklist is included in the manuscript as supplementary file.

## Supporting information

Supporting Information
